# Association between Diet and Adiposity in Adults: Influence of Sedentary Behavior Patterns

**DOI:** 10.3390/healthcare11081157

**Published:** 2023-04-18

**Authors:** Victor Spiandor Beretta, William Rodrigues Tebar, Catarina Covolo Scarabottolo, Leandro Dragueta Delfino, Bruna Thamyres Ciccotti Saraiva, Amanda Barbosa Santos, Ewerton Pegorelli Antunes, Isabella Cristina Leoci, Gerson Ferrari, Diego Giulliano Destro Christofaro

**Affiliations:** 1 Physical Education Department, School of Technology and Sciences, São Paulo State University (Unesp), Sao Paulo 19060-900, Brazil; 2 Centre of Clinical and Epidemiological Research, University Hospital, University of Sao Paulo, Sao Paulo 05403-000, Brazil; 3 Graduate Program in Movement Sciences, Physical Education Department, School of Technology and Sciences, São Paulo State University (Unesp), Sao Paulo 19060-900, Brazil; 4 Faculty of Health Sciences, Universidad Autónoma de Chile, Providencia 7500912, Chile

**Keywords:** dietary pattern, epidemiology, overweight, public health, sedentary behavior, nutrition

## Abstract

This study analyzed the influence of sedentary behavior (SB) on the association between dietary patterns and adiposity in community-dwelling adults. Eight hundred and forty-three adults (age: 56.5 ± 18.3 years) participated in this cross-sectional epidemiological study. Dietary patterns were evaluated using self-report questions regarding the weekly frequency of consumption of certain foods. Adiposity was determined using anthropometric measurements of weight, waist circumference, and height. SB was evaluated according to the time spent on screen devices. The usual physical activity level and socioeconomic status were considered confounding factors. Associations were determined using multivariate linear models with simultaneous adjustments for confounding variables. A statistical analysis indicated that fruit consumption was negatively related to the body mass index, regardless of the adjustment for SB domains. Red meat consumption was positively related to the body mass index, and fried food consumption was positively related to the waist-to-height ratio, regardless of the adjustment for SB domains. The consumption of fried food was positively associated with global and central adiposity after the adjustments for confounding factors and time spent on screen devices. We concluded that dietary habits are related to adiposity in adults. However, SB domains seem to influence the relationship between body adiposity and dietary habits, mainly regarding the consumption of fried foods.

## 1. Introduction

Over the past 50 years, the prevalence of obesity has increased to a pandemic dimension, becoming one of the biggest public health issues worldwide [1,2]. Obesity has been associated with several non-communicable diseases, such as cancer, diabetes mellitus, and cardiovascular impairments [1,3], and to an increased risk of mortality [4]. Obesity is considered an excessive accumulation of body fat that may affect an individual’s health [4]. Adults are defined as obese if they present a body mass index ≥30 kg/m^2^ [4,5]. In general, weight gain in obesity occurs through the accumulation of body adiposity due to an imbalance between energy intake and energy expenditure over time [6]. Previous studies considered the accumulation of body adiposity as global (determined by the body mass index) and central adiposity (determined by the waist-to-hip ratio or waist-to-height ratio) [7,8,9]. The main factors contributing to excessive energy intake and lower energy expenditure are unhealthy dietary patterns and physical inactivity, respectively [10,11,12,13].

Dietary patterns may directly impact quality of life and the risk of impaired health [13,14,15,16]. Adequate and balanced dietary habits, such as the consumption of fruits, vegetables, whole grains, and reduced-fat dairy products as well as the reduced consumption of red meat, have been associated with smaller gains in global and central adiposity [13,17,18]. On the other hand, unhealthy dietary habits (e.g., high intakes of fried food, fast food, sweets, and soft drinks) have been associated with obesity [19,20,21,22]. Furthermore, high consumptions of white bread and red meat/potatoes were associated with central and global adiposity in adults, respectively [17]. In addition to the risk of obesity, dietary habits seem to be associated with sedentary behavior in adults [23,24].

Sedentary behavior is defined as activities with low energy expenditure (≤1.5 metabolic equivalent task units) performed in sitting, lying, or reclining positions [25]. Spending a long time performing sedentary behavior has also been associated with the development of non-communicable diseases and the risk of mortality [26,27]. The mortality rate increased by almost 8% when the sitting time was more than eight hours a day [28].

In addition to the sitting time, the time spent on screen devices influences sedentary behavior, and as mentioned above, the dietary pattern seems to influence sedentary behavior [23]. While adequate dietary patterns increase the chance of being involved in physical activity and decrease the chance of using the computer (>4 h/day), unhealthy dietary patterns increase the chance of spending more time performing sedentary behavior [23]. However, controversial results have been reported, such as a lack of association between sedentary behavior and dietary habits in public school teachers [29]. Furthermore, sedentary behavior was associated with adiposity, regardless of diet [30]. In this sense, the association of specific dietary patterns with central and global obesity, as well as whether there is an influence of sedentary behavior patterns on this relationship, regardless of other confounding factors such as age, sex, socioeconomic status, and the habitual level of physical activity, is not clear.

Thus, we aimed to analyze the association of specific dietary habits with general and central adiposity in adults, considering the influences of different sedentary behavior domains and potential confounding factors.

## 2. Materials and Methods

### 2.1. Study Design and Participants

This was an epidemiological study with a cross-sectional design. The study was approved by the research ethics committee of São Paulo State University. The sample consisted of adults of both sexes. The inclusion criteria were (1) individuals aged ≥18 years that were (2) not institutionalized, (3) living in the urban area of Presidente Prudente, SP (a city in Brazil), and (4) not presenting physical limitations that made it impossible to stand up (e.g., wheelchair users and bedridden individuals). The exclusion criterion was not answering all the items of the questionnaire.

### 2.2. Sample Size Calculation

Presidente Prudente has an estimated population living in urban areas of 207,610, with a total of 176,124 individuals aged over 18, according to the Brazilian Institute of Geography and Statistics (IBGE). The sample size was calculated considering the outcome prevalence of 50% in epidemiological studies and α < 0.05, which resulted in at least 380 participants. A design effect correction of 50% was applied, considering the cluster sampling. In addition, predicting possible data losses, we increased the sample size by 10%, which resulted in a minimum required sample of 608 subjects. Participants were selected using a randomized process through a survey of all streets in Presidente Prudente, SP, which were divided according to the neighborhood, postal code, and geographic location into northern, southern, eastern, western, and central regions for data collection [31].

### 2.3. Data Collection

Data collection was carried out from March 2016 to August 2017 using randomized lists of the streets, considering the minimum sample needed in each region. On each of the selected streets, all households were visited. The number of participants was the same for each region (i.e., 608/5 = 122). Data collection was performed using face-to-face household interviews conducted by experienced evaluators (i.e., previously trained to apply questionnaires and minimize possible bias). The information was collected on tablets using a digital interface developed in the Open Data Kit (ODK) application [31]. Informed consent was obtained from all individual participants included in the study (N = 843).

### 2.4. Global and Central Adiposity

Global and central adiposity were considered as the dependent parameters of the present study. Global adiposity was interpreted using the body mass index [7,9]. This was determined using the objective measurements of weight and height, which were collected, respectively, using a Wiso^®^ digital scale (with a range of 0.1 to 180 kg) and a Sanny^®^ portable stadiometer (maximum height of two meters) with the scale in millimeters. Central adiposity was considered as the waist-to-height ratio, which was determined by dividing the waist circumference by the height. The abdominal circumference was determined at the midpoint between the iliac crest and the last rib using an inextensible anthropometric tape with a scale in millimeters (maximum length of two meters).

### 2.5. Dietary Habits

Dietary patterns were assessed using the self-reported weekly consumption of fruits, vegetables, cereals, dairy products, red meat, sweets, grains, fried foods, soft drinks, and snacks [29]. We quantified the frequency of weekly consumption (i.e., 0 to 7 days) of each of the mentioned foods. Dietary habits were considered as an independent parameter in the present study.

### 2.6. Sedentary Behavior

Sedentary behavior was measured using the time spent on screen devices and sitting time through an assessment of the number of hours per day an individual spends watching TV, using a computer, and using a cell phone or tablet during the week (0 to 7 days). Responses were categorized as (i) less than one hour (coded as 0); (ii) one hour or more but less than two hours (coded as 1); (iii) two hours or more but less than three hours (coded as 2); (iv) three hours or more but less than four hours (coded as 3); (v) four hours or more but less than five hours (coded as 4); and (vi) five hours or more (coded as 5). In addition, the total sitting time was determined using the self-reported time in hours and minutes.

### 2.7. Covariates

The variables of age, sex, socioeconomic level, and habitual physical activity were included as covariates in the present study (i.e., confounding parameters). The socioeconomic level was calculated according to the Brazilian criteria for economic classification [32], which consider the educational level and the numbers of specific rooms and consumer goods at home. The information was categorized into high (classes A and B1), medium (classes B2 and C1), and low (classes C2, D, and E) socioeconomic levels.

The level of habitual physical activity was assessed using the Baecke questionnaire [33], which is composed of questions about the frequency, duration, and intensity of physical activities performed in three different settings (leisure time/commuting, occupation, and sports practice). The Baecke questionnaire was previously validated for Brazilian adults against accelerometry [34] and provides a dimensionless score ranging from 1 to 5 for each assessed domain. The sum of the three settings corresponds to the total physical activity score.

### 2.8. Statistical Analysis

The statistical analyses were performed using SPSS (version 20.0, SPSS Inc., Chicago, IL, USA), and the significance level was maintained at 0.05. The descriptive characteristics of the sample are presented as means and standard deviations. The Pearson correlation test was performed to analyze the bivariate relationships of dietary habits with global and central adiposity and with the sedentary behavior domains. Linear regression models were created to analyze the magnitude of the association between dietary habits and adiposity (model 1) and the magnitude this association with the addition of the confounding factors of sex, age, socioeconomic level, and habitual physical activity (model 2). In addition to the sex, age, socioeconomic level, and habitual physical activity, two other models considered sedentary behavior, including sitting time (model 3) and the time spent on screen devices (model 4).

## 3. Results

### 3.1. Descriptive Characteristics

The descriptive characteristics of the sample are presented in Table 1. The sample was mainly composed of women (61.7%). In total, 176 individuals (21%) were classified as having a high socioeconomic level, 619 (73%) were classified as having a medium socioeconomic level, and 48 (6%) were classified as having a low socioeconomic level (Table 1).

### 3.2. Correlations between Dietary Habits and Global and Central Adiposity

**Figure 1:**
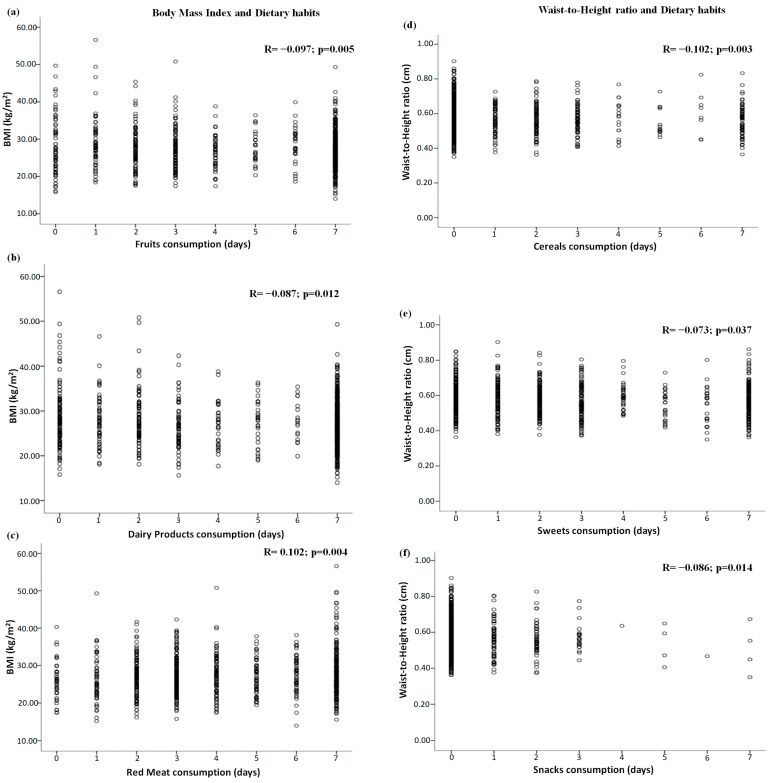
Significant correlations between dietary habits and global and central adiposity: (**a**–**c**) correlations between global adiposity (i.e., body mass index) and fruit, dairy product, and red meat consumption, respectively, and (**d**–**f**) correlations between central adiposity (i.e., waist-to-height ratio) and cereal, sweet, and snack consumption, respectively. Note: BMI = body mass index.

The significant correlations between dietary patterns and adiposity are shown in Figure 1. Pearson’s correlation test indicated a small negative correlation between the body mass index and fruit and dairy product consumption (all *p* < 0.02), and a small positive correlation between the body mass index and red meat consumption (*p* = 0.004) (Figure 1a–c, respectively). Small negative correlations were demonstrated between the waist-to-height ratio and cereal, sweet, and snack consumption (all *p* < 0.04) (Figure 1d–f, respectively).

### 3.3. Correlations between Dietary Habits and Sedentary Behavior Domains

**Figure 2:**
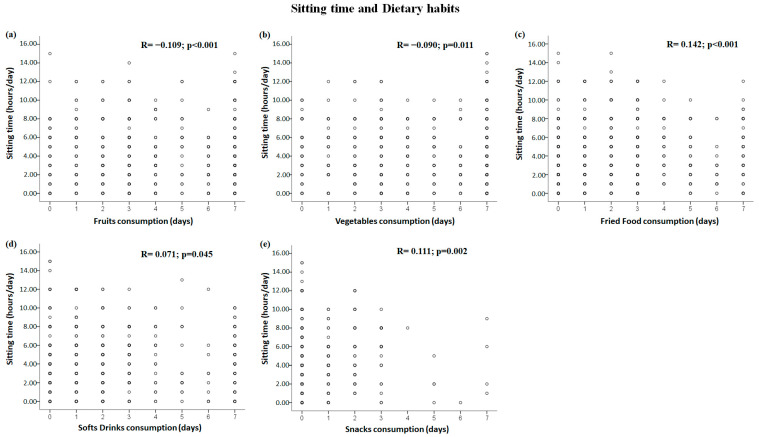
Significant correlations between dietary habits and sitting time domain: (**a**–**e**) correlations between sitting time and fruit, vegetable, fried food, soft drink, and snack consumption, respectively.

**Figure 3:**
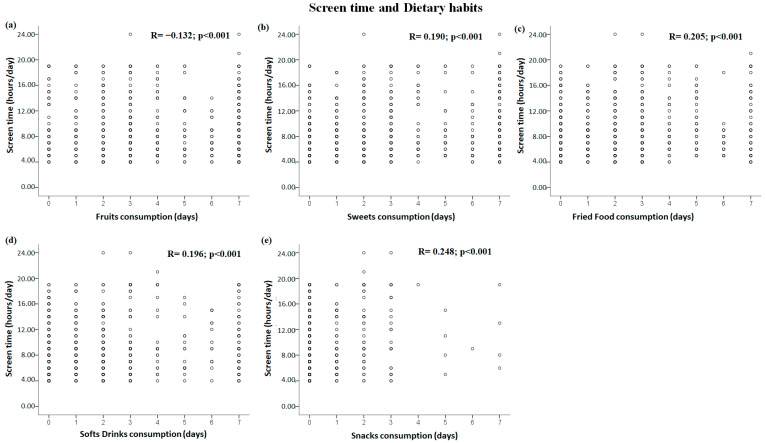
Significant correlations between dietary habits and screen time domain: (**a**–**e**) correlations between screen time and fruit, sweets, fried food, soft drink, and snack consumption, respectively.

The significant correlations between dietary patterns and sedentary behavior are shown in Figure 2 for the sitting time domain and Figure 3 for the screen time domain. Fruit consumption was negatively correlated with sitting time and screen time (all *p* < 0.006) (Figure 2a and Figure 3a), and vegetable consumption was negatively correlated with sitting time (*p* = 0.011) (Figure 2b). Sweet consumption was positively correlated with screen time (*p* < 0.001) (Figure 3b). In addition, positive correlations were demonstrated between all sedentary behavior domains (i.e., sitting time and screen time) and fried food (Figure 2c and Figure 3c), soft drink (Figure 2d and Figure 3d), and snack consumption (all *p* < 0.05) (Figure 2e and Figure 3e).

### 3.4. Associations between Dietary Habits and Global Adiposity

The linear associations between body mass index (i.e., global adiposity) and dietary patterns are shown in Table 2. Multiple adjusted models (i.e., including age, sex, socioeconomic condition, habitual physical activity, and sedentary behavior domains) showed that the body mass index was related to lower fruit and dairy product consumption (all *p* < 0.007). In addition, all adjusted models indicated that the body mass index was related to higher red meat consumption (all *p* < 0.005). Models 3 (*p* = 0.044) and 4 (*p* = 0.049) demonstrated that the body mass index was related to lower sweet consumption. In addition, model 4, which included screen time, indicated that the body mass index was related to higher fried food consumption (*p* = 0.040).

### 3.5. Associations between Dietary Habits and Central Adiposity

The linear associations between the waist-to-height ratio (i.e., central adiposity) and dietary patterns are shown in Table 3. All adjusted models demonstrated that the waist-to-height ratio was related to lower fruit, cereal, and dairy product consumption (all *p* < 0.007). Furthermore, all adjusted models showed that the waist-to-height ratio was related to higher red meat and fried food consumption (all *p* < 0.04). In addition, when adjusted for the confounding factors and sedentary behavior domains, the negative correlations of sweet and snack consumption with central adiposity were no longer evident (i.e., a lack of statistical significance; *p*-value range = 0.08–0.686).

## 4. Discussion

The present study showed that global adiposity was associated with lower fruit and dairy product consumption and with higher red meat consumption, regardless of sedentary behavior and confounding factors. Sedentary behavior influenced the association of global adiposity with fried food and sweet consumption. In addition, central adiposity was associated with lower fruit, cereal, and dairy product consumption and higher red meat and fried food consumption regardless of sedentary behavior. After adjustment for sedentary behavior, the inverse association between central adiposity and vegetable consumption and the association of global and central adiposity with higher fried food consumption became significant. The association of central adiposity with sweet and snack consumption lost significance in the adjusted models.

The negative association between global adiposity and dairy product consumption observed in the present study is supported by previous findings [35,36] and was observed in the present study, even after multiple adjustments for confounders, including sedentary behavior patterns. The consumption of dairy products, mainly milk, was associated with lower global adiposity (i.e., body mass index) in a very large population (*n* = 39,640) [36]. Several possible biological explanations for the positive influence of dairy product consumption on adiposity have been reported [35]. Low calcium intake affects calcium-dependent mechanisms that could increase fat synthesis and decrease lipolysis [35]. On the other hand, adequate calcium intake, combined with normal protein intake, seems to increase fecal fat excretion [37]. The magnesium content of milk may also improve insulin sensitivity [35], as demonstrated by the decrease in insulin resistance in overweight adults, which could reduce the risk of type 2 diabetes mellitus and cardiovascular disease [38].

An inverse association of global and central adiposity with fruit consumption was observed in the present study. It has previously been reported that a dietary pattern that includes large amounts of fruit and low amounts of red meat seems to impact weight loss and maintenance [39,40,41,42]. Although controversial results exist [43], in general, fruit consumption contributes to weight loss or maintenance [44]. The frequent consumption of fruits contributes to higher fiber intake, which promotes a satiety effect, contributes to intestinal motility [40], and precludes the consumption of energy-dense foods [39], leading to a decrease in fat intake that could impact body adiposity [40]. On the other hand, global and central adiposity were associated with higher red meat consumption. A dietary pattern with higher red meat has previously been associated with global and central adiposity, mainly due to the higher fat content and energy intake [45,46]. Although these associations remained significant, even in the multiple adjusted statistical models, the high energy intake combined with excessive time with low energy expenditure in sedentary activities of daily life substantially contributed to the positive caloric imbalance and the consequent weight gain and increase in adiposity [13].

The crude analysis demonstrated an unexpected inverse association of snacks and sweets with central adiposity in the present study. However, the association lost significance after adjustment for covariates and sedentary behavior patterns. The consumption of sweets has been associated with higher sedentary behavior [47] and insufficient physical activity levels [48], reflecting the potential role of lifestyle habits in food consumption and daily energy expenditure, which could explain, at least in part, the annulment of this association. In addition, the consumption of fried foods became associated with global and central adiposity after adjustment for covariates and sedentary behavior patterns in the present study. A previous study reported that the combination of sedentary behavior and fatty and fried food consumption resulted in a positive multiplicative interaction for increases in obesity [49]. Frying foods modifies their composition, as they lose water and absorb fat during preparation. This procedure increases the calories per gram (energy density) and increases the crispness and palatability, which could lead to excessive consumption and, consequently, higher caloric and fat intake [50].

As a specific characteristic of this study sample, we observed elevated screen time, with an average of over 8 h/day, which may have influenced the results. A long time spent on screen devices has been associated with long-term weight gain. Adults who spent more time on screen devices, such as watching TV (i.e., 24 h/day), presented an increased probability of being overweight [51]. These individuals demonstrated higher consumption of added sugar and total fat when compared to those who watched television for less than 1 h/day [51]. In addition to the sugar intake, a longer time on screen devices was associated with a high frequency of consumption of foods with low nutritional quality, such as fast foods and fried foods [52], and with higher soft drink consumption [53]. A possible factor that contributes to the association between unhealthy foods and screen time devices is food advertisements, which were shown to influence the consumption of fried foods, snacks, sweets, and fast foods [54]. A meta-analysis reported that the excessive consumption of unhealthy foods has been associated with weight gain of 0.12 to 0.22 kg per year [55]. Thus, sedentary behavior patterns, mainly excessive time in front of screens, seem to influence unhealthy food consumption, which substantially contributes to overweight and obesity [52].

As positive aspects, we observed that this study sample had a high frequency of consumption of grains (practically every day of the week) and vegetables (more than 5 days a week). The Brazilian population traditionally consumes rice, beans, and meat as staple foods [56], and the frequent access to these foods might have been affected by regional agriculture and livestock. These specific characteristics of the sample may have mitigated the association of the consumption of grains and vegetables with adiposity levels.

Among the limitations of the present study, it is important to highlight the self-reported assessment of habitual physical activity and sedentary behavior, which is susceptible to the bias of memory and classification. In addition, food consumption was not investigated regarding the daily portions, which may substantially impact the caloric intake and, consequently, the relationship with adiposity levels. Another study limitation is the cross-sectional design, which does not allow the establishment of the direction of causality (i.e., whether food consumption caused overweight and how long the individual has had this type of lifestyle). Furthermore, we considered screen time regardless of the type of device (i.e., TV, cell phone, computer, and tablet). However, the type of screen device seems to influence the association with dietary patterns [49]. Thus, future studies should consider the influence of different screen time devices in the association of dietary patterns and adiposity. In addition, future studies should include objective measurements of physical activity and sedentary behavior (i.e., accelerometers) to advance the knowledge regarding the influence of these confounding factors in the relationship between dietary habits and adiposity. Accelerometers have been used to identify physical activity during waking hours (at least 10 h per day) for at least four days. The signal of the accelerometer (i.e., the accelerometry of the minutes in activity) is converted into counts per minute, and there are validated cutoff points for the classification of physical activity intensity and sedentary behavior [57].

Despite the study’s limitations, our results extend the current literature by providing a greater understanding regarding the influence of sedentary behavior on the association between an unhealthy diet (i.e., high fried food consumption) and global and central adiposity in a large sample. It should be highlighted that our study includes analyses adjusted for potential confounding factors. Strategies to reduce sedentary behavior and to reduce the consumption of foods rich in sugars and fats could be effective in controlling body weight in adults and in addressing the obesity epidemic.

## 5. Conclusions

Dietary patterns were related to global and central adiposity in adults. However, sedentary behavior only appeared to influence the relationship between adiposity and dietary habits, mainly regarding the consumption of sweets and fried foods. This information may be helpful to guide further studies and health policies for the promotion of healthy eating to control adiposity parameters in the adult population.

## Figures and Tables

**Table 1:** Descriptive characteristics of the sample.

Parameters	Mean ± std
Age, years	56.53 ± 18.32
Sex (male/female)	324/519
Body Mass Index, kg/m^2^	27.3 ± 5.8
Waist-to-Height Ratio, cm	0.6 ± 0.1
Sitting Time, hours/day	3.8 ± 3.0
Screen time, hours/day	8.5 ± 3.7
Habitual Physical Activity (Baecke score)	11.7 ± 2.8
Socioeconomic Level, score	30.12 ± 9.7
Fruits, days/week	4.6 ± 2.6
Vegetables, days/week	5.1 ± 2.4
Cereals, days/week	1.4 ± 2.2
Dairy Products, days/week	4.6 ± 2.8
Grains, days/week	6.7 ± 1.0
Red Meat, days/week	4.0 ± 2.2
Sweets, days/week	2.8 ± 2.5
Fried Foods, days/week	2.0 ± 2.1
Soft Drinks, days/week	1.7 ± 2.1
Snacks, days/week	0.3 ± 0.9

Notes: std = standard deviation.

**Table 2:** Associations between body mass index (global adiposity) and dietary patterns in adults.

	Body Mass Index (Global Adiposity)			
Consumption Measured in Days	Model 1 β (95% CI)	Model 2 β (95% CI)	Model 3 β (95% CI)	Model 4 β (95% CI)
Fruits	**−0.22 (−0.37; −0.06)**	**−0.26 (−0.43; −0.01)**	**−0.28 (−0.46; −0.11)**	**−0.26 (−0.43; −0.10)**
Vegetables	−0.15 (−0.31; 0.02)	−0.14 (−0.31; 0.03)	−0.14 (−0.31; 0.04)	−0.14 (−0.31; 0.03)
Cereals	−0.17 (−0.35; 0.05)	−0.15 (−0.33; 0.04)	−0.17 (−0.36; 0.02)	−0.15 (−0.33; 0.04)
Dairy Products	**−0.18 (−0.32; −0.04)**	**−0.20 (−0.35; −0.06)**	**−0.24 (−0.39; −0.09)**	**−0.20 (−0.35; −0.06)**
Grains	0.07 (−0.33; 0.46)	0.07 (−0.33; 0.46)	−0.04 (−0.45; 0.38)	0.07 (−0.33; 0.46)
Red Meat	**0.27 (0.09; 0.45)**	**0.27 (0.09; 0.45)**	**0.28 (0.01; 0.47)**	**0.27 (0.09; 0.45)**
Sweets	−0.13 (−0.29; 0.02)	−0.15 (−0.31; 0.01)	**−0.17 (−0.33; −0.01)**	**−0.16 (−0.31; −0.01)**
Fried Foods	0.15 (−0.04; 0.34)	**0.21 (0.01; 0.41)**	0.17 (−0.04; 0.38)	**0.21 (0.01; 0.41)**
Soft Drinks	−0.02 (−0.20; 0.17)	−0.02 (−0.21; 0.17)	0.01 (−0.20; 0.20)	−0.02 (−0.21; 0.18)
Snacks	−0.38 (−0.84; 0.07)	−0.33 (−0.79; 0.13)	−0.45 (−0.93; 0.03)	−0.34 (−0.81; 0.13)

Notes: Model 1 = Bivariate analysis (unadjusted model); model 2 = adjusted by age, sex, socioeconomic level, and habitual physical activity; model 3 = adjusted by model 2 + sitting time; and model 4 = adjusted by model 2 + screen time, CI = confidence interval. Bold values indicate a statistically significant association (*p* < 0.05).

**Table 3:** Associations between waist-to-height ratio (central adiposity) and dietary patterns in adults.

	Waist-to-Height Ratio (Central Adiposity)			
Consumption Measured in Days	Model 1 β (95% CI)	Model 2 β (95% CI)	Model 3 β (95% CI)	Model 4 β (95% CI)
Fruits	0.001 (−0.002; 0.003)	**−0.003 (−0.006; −0.001)**	**−0.004 (−0.006; −0.001)**	**−0.004 (−0.006; −0.001)**
Vegetables	−0.002 (−0.004; 0.001)	−0.002 (−0.005; 0.000)	−0.002 (−0.005; 0.000)	**−0.003 (−0.005; −0.001)**
Cereals	**−0.004 (−0.007; −0.001)**	**−0.004 (−0.006; −0.001)**	**−0.004 (−0.007; −0.001)**	**−0.004 (−0.007; −0.001)**
Dairy Products	−0.001 (−0.003; 0.001)	**−0.003 (−0.005; −0.001)**	**−0.003 (−0.006; −0.001)**	**−0.003 (−0.005; −0.001)**
Grains	0.006 (−0.001; 0.012)	0.004 (−0.002; 0.010)	0.001 (−0.005; 0.007)	0.004 (−0.002; 0.009)
Red Meat	0.002 (−0.001; 0.004)	**0.003 (0.001; 0.006)**	**0.003 (0.001; 0.006)**	**0.003 (0.001; 0.006)**
Sweets	**−0.003 (−0.005; −0.001)**	−0.002 (−0.004; 0.001)	−0.002 (−0.005; 0.001)	−0.002 (−0.004; 0.000)
Fried Foods	−0.001 (−0.004; 0.002)	**0.004 (0.001; 0.007)**	**0.003 (0.001; 0.006)**	**0.004 (0.001; 0.007)**
Soft Drinks	−0.002 (−0.005; 0.001)	0.000 (−0.002; 0.003)	0.001 (−0.002; 0.004)	0.000 (−0.002; 0.003)
Snacks	**−0.009 (−0.015; −0.002)**	−0.001 (−0.008; 0.005)	−0.003 (−0.010; 0.003)	−0.001 (−0.008; 0.005)

Notes: Model 1 = bivariate analysis (unadjusted model); model 2 = adjusted by age, sex, socioeconomic level, and habitual physical activity; model 3 = adjusted by model 2 + sitting time; and model 4 = adjusted by model 2 + screen time, CI = confidence interval. Bold values indicate a statistically significant association (*p* < 0.05).

## Data Availability

All data are available from the corresponding author upon reasonable request.
